# News and Notes

**Published:** 1899-03

**Authors:** 


### NEWS AND NOTES.

---

DR. C. J. VAUGHAN has been elected county physician for Fulton county.

---

DRS. GASTON & GASTON have removed their offices to the Prudential building.

---

DR. ALFRED F. GRIGGS has removed his office to the Prudential building, rooms 601-602.

---

DRS. CHILDS & CHAMPION have removed their offices to 701, 702, 703 Prudential building.

---

DR. E. C. DAVIS and Dr. J. D. Cromer have removed their offices to the English-American building.

---

THE following are the officers of the Medical Board of Grady Hospital for 1899: President, Dr. V. O. Hardon; vice-president, Dr. J. G. Earnest; secretary and treasurer, Dr. Dunbar Roy.

---

THE Grady Hospital Training School for Nurses has now been regularly organized and incorporated under the laws of the State. The president of the Training School is Dr. Jas. B. Baird, and the secretary Dr. Dunbar Roy.

---

ON the evening of January 31st the Medical Board of Grady Hospital held its annual banquet at the Kimball house. The newly elected president for the coming year, Dr. V. O. Hardon, presided. The trustees of the hospital were present as invited guests and a most enjoyable evening was spent.

---

A CASE of almost sudden death, due to the standing in the way of the sudden escape of a large amount of Pintsch gas, occurred recently in Atlanta. Pintsch gas is that which is used in lighting the Pullman cars. The workman was attempting to open a pipe which had become stopped, when it suddenly opened, and he received the full escape. He was rendered unconscious from the shock, and even after its return, he suffered with the most violent spasms and nausea.

---

DR. ARNOLD, of Rome, Ga., has been fined $75.00 for not obeying a subpœna from the superior court of that city. Dr. Arnold was summoned as a witness, but refused to answer the same, and the judge made this fine for contempt of court.

---

WE have received the first annual report of the University Hospital at Kansas City, Mo. It is very neatly gotten up.

---

DR. GEO. ROHE, of Baltimore, one of the most prominent physicians in that section of the country, recently died in New Orleans.

---

A NEW monthly journal has made its first appearance. It is the *Southern Medical Journal*, and is published in Kinston, N. C., by Dr. J. W. P. Smithwick.

---

MR. C. B. KIRKLAND, who, for a number of years, has been connected with the advertising department of Parke, Davis & Co., has severed his connection with the latter firm to accept a position with the J. C. Ayer & Co., of Lowell, Mass.

---

THE Knights of Pythias propose to build a sanitarium at Hot Springs, Ark., at a cost of $500,000, of which $12,000 has been collected. The members of the society in the United States are to be assessed one dollar each for the realization of the project.

---

THE number of students enrolled at the Medical School in Paris is 4,495. The faculty includes 351 male foreign students, of whom 16 are Swiss, 15 Germans, 60 Roumanians, 67 Ottomans, and 66 Russians. There are also 87 female foreign students, of whom 83 are Russians.

---

GUY'S HOSPITAL has received an anonymous gift of $6,000 for the endowment of medical research, as the result of an address by Mr. Balfour, leader of the House of Commons, on the subject of research, on the occasion of the distribution of prizes at the medical school of Guy's Hospital.

---

OVER $100,000 has been subscribed for the Waring Fund. It will be invested and the income be given to the widow and daughter of the late Colonel George Waring. At their death it will revert to Columbia University to found a professorship to be known as the Waring professorship.

---

IN Paris in 1900 a "Biological Institute" is to be built next door to the Pasteur Institute. The expense will be covered by a sum of $400,000 recently bequeathed for this purpose. It will contain laboratories, lecture theaters, and a hospital, where diphtheria, lockjaw, and other diseases will be treated on Dr. Roux's system.

DR. H. V. WURDEMANN of Milwaukee, who has been associate editor of the *Annals of Ophthalmology*, has accepted the position of editor-in-chief, vice Dr. Casey A. Wood of Chicago, resigned. Dr. Wood will retain an interest in the publication, and will have charge of the department of Italian literature.

---

DODD, in the *Lancet* for December 6, 1898, reports a severe case of hemophilia, which was almost moribund from exsanguination when he began the administration of oxygen, which immediately checked the collapse, caused the vomiting and bleeding to cease, and eventually brought about complete recovery.

---

WITH the December issue the *Ohio Medical Journal* ceased its publication. The reasons, as given by its editor, are: "The object for which the journal was established has been fulfilled, the multiplication of monthly periodicals within the last few years, and the inability of the present manager to longer devote his time to its publication."

---

FRANCIS A. MOCKTON in the *British Medical Journal* for November 19, 1898, reported a number of cases of diabetes mellitus successfully treated by intestinal antiseptics, chiefly sodium sulphocarbolate and boric acid. The improvement was attributed to the checking of the fermentation in the intestines, which is thought to produce excessive quantities of glucose in this condition.

---

IN order to prevent the yellowing of the coatings in water-closets, stables, etc., where the exhalations are so strong that the paint turns yellow in twenty-four hours, no white lead paint should be employed, says the *Deutsche Malerzeitung*. Use only zinc white with a suitable mixing color, thinned with turpentine and water. It is true that zinc white is also attacked by strong exhalations, but not in such a degree as white lead which even under wood-color becomes black and spotted in a short time.

---

THE Ninth International Congress of Ophthalmology will be held at Utrecht from August 14 to 18, 1899. The scientific work of the Congress will be divided among three sections, as follows: (1) Anatomy, Pathological Anatomy and Bacteriology; (2) Optics and Physiology; (3) Clinical and Operative Methods. The communications and discussions will be presented in either French, English, or German. Dr. D. Argyle Robertson, President of the Eighth Congress, is Chairman of the Committee on Organization.

---

THE medical profession of Alabama is certainly to be commended for its progressiveness. This body of men is attempting to have laws passed for the good of the people at large. A strong movement is on foot by the physicians all over the State to have the legislation passed making vaccination compulsory upon all school children. The profession of Birmingham is also endeavoring to have a city law passed prohibiting spitting in public buildings and public places of an enclosed nature. Georgia would do well to take up the same subject.

---

DEATH OF PRESIDENT PARKE.—The head of the corporation, Parke, Davis & Co., died in California in February. Mr. Parke was born December 13th, 1827. After reaching manhood he was in various business until about 1867, when he formed that which finally became the present great corporation. He has always been president of the company, which was incorporated in 1876. Personal friends of Mr. Parke tell us that he was a most excellent and lovable gentleman. His company is noted for its courtesy to and appreciation of medical journals, and well reflects the character of Mr. Parke.

WE learned from the *Cleveland Journal of Medicine* for January, that Harriet O. Evans, a "Christian Scientist" of Cincinnati, complacently allowed Thomas McDowell to die of typhoid fever without any treatment except the much-vaunted prayer of these peculiar people. Under the energetic initiative of Dr. Charles A. L. Reed of Cincinnati, who is a member of the State Board, she was prosecuted for practicing medicine without a license. The police court jury is reported to have employed just twenty minutes in deciding that she was guilty as charged. The case has been appealed, ot course, but it is a most excellent beginning. The thanks of the medical profession of the State, says the *Cleveland Journal,* are due to Dr. Reed for having shown in this and many other cases what a sincere and energetic member of the board can accomplish.

---

AT the Columbus meeting of the American Medical Association in June, in addition to their regular programs, the Section in Ophthalmology and the Section in Laryngology and Otology will devote the morning of the second day, June 7th, to a joint meeting, under the chairmanship of Dr. Casey A. Wood of Chicago, and of Dr. Emil Mayer of New York. The subject for discussion will be "The Relation of Ocular Diseases to Affections of the Nose and Neighboring Cavities." Four papers are to be read on this subject, by invitation, as follows: Dr. Charles Stedman Bull of New York, on "Some Points in the Symptomatology, Pathology, and Treatment of Sinuses Adjacent and Accessory to the Orbit"; Dr. D. Bryson Delavan of New York, on "Nasal Stenoses in their Relation to Ocular Disturbances"; Dr. Joseph A. White of Richmond, Va., on "Eye Troubles Attributable to Nasopharyngeal and Aural Disturbances"; Dr. J. H. Bryan of Washington, D. C., on "Diseases of the Accessory Sinuses in their Relation to Diseases of the Eye." The general discussion will be on the main question.

IN a kissing contest, says the *Medical Record*, for £5 a side, which has just been declared off in a small town in Lancashire, the challenger sank exhausted at the seven hundredth smack, his opponent having scored eighteen hundred in the hour. Over in Germany at about the same time a young lover wrote to his sweetheart that he sent her ten thousand kisses. She sent back word that that was all well enough on paper, but that he had not the nerve to perform the feat in a truly manly way. The challenge was likewise accepted, and ten hours' time was allowed, with brief intervals for malt refreshments. At the end of the first hour the score was two thousand, and the condition of both active and passive participant good. One thousand more were added in the second hour, but at the seven hundred and fiftieth kiss on the third round the young man's lips became paralyzed and he lost consciousness. This fate, which some might consider merited, should prove a warning to promiscuous kissers, as well as to those who osculate with persistence. It is not paralysis alone, but insanity, which may follow such efforts.

---

AMERICAN ACADEMY OF MEDICINE—*Preliminary Program for the Columbus Meeting.*—The Committee on Program for the Columbus meeting is much encouraged by the promises made to present papers. It is desired to restrict the number of papers in order to afford ample time for discussions; hence it is requested that any one who is purposing to present a paper at the next meeting promptly notify a member of the committee of the fact.

At the meeting at Denver two subjects for discussion were suggested, and the comments on the medical service of the army and navy during the war with Spain furnished a third timely topic. There will be consequently, three special topics presented to the academy for its discussion, the discussion to be opened by a series of carefully prepared papers.

Thus far the following papers have been promised, arranged under the special topic to be discussed.

*Specialism in Medicine.*

1. "The Ethics of Specialism," by Dr. A. L. Benedict, Buffalo.

2. "How far has Specialism Benefited the Ordinary Practice of Medicine," by Dr. L. Duncan Bulkley of New York.

3. "The Relation of Specialism to General Medicine," by Dr. Solomon Solis-Cohen of Philadelphia.

4. "The Specialties and Commercialism," by Dr. H. Bert Ellis of Los Angeles.

5. Subject to be announced, by Dr. G. Hudson Makuen of Philadelphia.

6. "Specialists and Therapy," by Elmer Lee, M.D., of New York.

7. "Specialism in its Relation to other Branches of Medicine," by Dr. G. W. McCaskey of Fort Wayne, Ind.

*Advertising and the Medical Profession.*

1. "Medical Advertising," by Robert H. Babcock, of Chicago.

2. "The Ethics of Advertising Applied to the Medical Profession," by Dr. A. Ravogli of Cincinnati.

3. Subject to be announced, by Dr. J. A. Lichty of Clifton Springs, N. Y.

*The Medical Service of the Army and Navy.*

1. "Aspects of the Late War from a Naval Surgeon's Standpoint," by Dr. W. C. Braisted, U. S. N.

2. Subject to be announced, by Dr. G. G. Groff, now in Puerta Rico.

3. "What Should be Aimed at in a Reorganization of the National Guard System so far as its Medical Department is Concerned?" by Dr. C. B. Nancrede, late of the U. S. V., of Ann Arbor, Mich.

4. "The Relation of the Medical Profession to the Army and Navy as Developed in the Spanish-American War," by Dr. James A. Pilcher, U. S. A.

*Miscellaneous Papers.*

1. "Remarks on Hospital Organization, with Special Reference to Continuous Service," by Dr. J. C. Wilson of Philadelphia.

*Papers on Subjects Not Yet Furnished.*

1. By Dr. Edward Jackson of Denver, President's Address.
2. By Dr. J. R. Smith, U. S. A. Retired.
3. By Dr. V. C. Vaughan of Ann Arbor.
4. By Dr. C. T. McClintock of Detroit.
5. By Dr. A. J. Puls of Milwaukee.

An effort will be made to issue a circular containing a brief sypnosis of the papers to be presented, long enough before the meeting to enable those who attend to acquaint themselves with the trend of the papers, and to prepare themselves for a more profitable discussion.

The Secretary has one request to make in connection with the Columbus meeting. Experience has shown that it is helpful to know who are expecting to attend the meeting. If, for example, near the time for the meeting, he receives some special information of value to those who may attend, he is able to communicate this, while a general circular might be impracticable. The request is that you at once send him word, if you are planning to go. Of course, if you have already done so, this message is not for you.—*Bulletin of the American Academy of Medicine.*

---

***NOTICE.—This JOURNAL and THE INTERNATIONAL JOURNAL OF SURGERY one year for $1.00 cash, to new subscribers. No out-of-town checks accepted unless 10c. is added for cost of cashing.***

